# Evaluating the link between *DIO3*-FA27 promoter methylation, biochemical indices, and heart failure progression

**DOI:** 10.1186/s13148-024-01668-0

**Published:** 2024-04-24

**Authors:** Yan Qi, Xiangchao Meng, Jing Li, Aoyue He, Jie Hao, Xu Zhao, Ruonan Zhao, Rongrong Chen, Rongqiang Zhang

**Affiliations:** 1 Department of Epidemiology and Health Statistics, School of Public Health, Shaanxi University of Chinese Medicine, Xianyang, 712046 Shaanxi China; 2 Public Health Department, Jinan Children’s Hospital, Jinan, 25000 Shandong China

**Keywords:** Heart failure, Methylation, Biochemical indices, CpG

## Abstract

**Background:**

Heart failure (HF) is a disease that poses a serious threat to individual health, and DNA methylation is an important mechanism in epigenetics, and its role in the occurrence and development of the disease has attracted more and more attention. The aim of this study was to evaluate the link between iodothyronine deiodinase 3 promoter region fragment FA27 (*DIO3*-FA27) methylation levels, biochemical indices, and HF.

**Results:**

The methylation levels of *DIO3*-FA27_CpG_11.12 and *DIO3*-FA27_CpG_23.24 significantly differed in HF patients with different degrees. Multivariate logistic regression analysis indicated that the relative HF risk in the third and fourth quartiles of activated partial thromboplastin time and fibrin degradation products. The results of the restricted cubic spline model showed that the methylation levels of *DIO3*-FA 27_CpG_11.12 and *DIO3*-FA 27_CpG_23.24 were associated with coagulation indicators, liver function, renal function, and blood routine.

**Conclusions:**

Based on the differential analysis of CpG methylation levels based on *DIO3*-FA27, it was found that biochemical indicators combined with *DIO3*-FA27 promoter DNA methylation levels could increase the risk of worsening the severity classification of HF patients, which provided a solid foundation and new insights for the study of epigenetic regulation mechanisms in patients with HF.

**Supplementary Information:**

The online version contains supplementary material available at 10.1186/s13148-024-01668-0.

## Background

Heart failure (HF) is a global health and financial concern [1]. Due to population growth and aging, the total number of patients with HF continues to rise, accounting for 64.3 million people living with HF globally. In developed countries, the known HF prevalence is estimated to be 1–2% of the general adult population [2, 3]. Moreover, epigenetics is defined as “a stable heritable phenotype that does not depend on changes in DNA sequences, is a result of chromosomal alterations.” DNA methylation, changes in histone structure, and regulation of genes by microRNAs are important vectors of epigenetic information. Among them, DNA methylation is one of the important epigenetic modifications, which has a profound impact on genome stability, transcription, and development [4]. In 2021, research reports on the association between DNA methylation and HF appeared for the first time, opening up a new idea for HF diagnosis and treatment [5, 6].

Selenium is one of the essential trace elements for the human body and plays a vital role in human health. Recently, people have distinguished its nutritional value from its toxicity [7]. Selenocytes have an antioxidant effect due to the oxidized state of the selenium atoms [8]. Deiodinase (DIO) of selenoproteins is an oxidoreductase, although they act on thyroid hormones rather than peroxides [9]. There is a clear association between DNA methylation and cardiovascular illness, as shown by research on several disorders where the same CpG sites were found to be differentially methylated [10, 11]. It also lends credence to the theory that some CpG methylation is probably disease relevant. The analysis of the CpG-annotated genes provides more evidence for this. Conversely, genes that they have in common are associated with more generic words and diseases related to cardiovascular disease [12].

Studies have contrasted the metabolic characteristics of nonischemic HF etiologies with those of ischemic cardiomyopathy, in which the heart displays hypermethylation and oxidative pathway gene silencing [13]. Nevertheless, it is unclear how DNA methylation affects the transcriptional processes shared by all HF etiologies and how these modifications differ from those seen in human hearts that are not failing. Whether selenium deficiency in patients with HF is only a marker of worsening disease severity or a causative factor in HF development and progression remains to be clarified. In addition, the pathophysiology and molecular mechanisms affected by selenium deficiency are underrepresented in current HF studies and require more attention [14].

The *DIO3* gene plays a central role in thyroid hormone metabolism. The multiple promoter regions of *DIO3* are worthy of exploring the relationship between their CpG methylation levels and HF. *DIO3*-FA27 has the most CpGs sites and more dense. Herein, we determined CpGs and CpG-SNPs on the *DIO3*-FA27 promoter region. To measure the aforementioned CpG methylation levels and determine the CpG-SNP genotypes, matrix-assisted laser desorption/ionization time-of-flight mass spectrometry (MALDI-TOF–MS) was utilized. The potential correlation of CpG methylation levels in patients with HF with those in different genotypes was analyzed. This study aimed to identify differential CpG sites and CpG-SNPs of *DIO3*-FA27 in patients with HF.

Nonetheless, prior research has mostly concentrated on conventional risk variables for long-term heart failure prediction. Patients in hospitals require short-term prediction in order to streamline the process of managing and triaging many patients, as emergencies frequently arise during this time. However, some laboratory results, like total bilirubin (TBIL), prothrombin time (PT), activated partial thromboplastin time (APTT), blood urea nitrogen (BUN), glucose, and serum creatinine (SCR), may be useful predictors of HF and could enhance the model’s predictive accuracy [14]. Therefore, research is still needed on the problem of establishing prediction models and discovering new laboratory predictors for short-term HF, as it has not yet been thoroughly explored in the literature.

## Methods

### Study population

Herein, we collected 20 patients diagnosed with HF in the Affiliated Hospital of Shaanxi University of Traditional Chinese Medicine from January 2023 to June 2023. Furthermore, a control group consisting of 20 healthy individuals with normal cardiac function was chosen from the hospital’s physical examination center. The diagnosis of the case group and the control group was based on the “2018 Chinese Guidelines for the Diagnosis and Treatment of Heart Failure.” The institutional review board of Medical Ethics Committee of Ningxia Hui Autonomous Region People’s Hospital (ZDYF-046; 2021) accepted this study, and the patient completed an informed consent form. The frequency matching of both groups of study subjects was used to ensure that the age, sex, and other indicators of the case and control groups were comparable.

### Quantitative DNA methylation analysis on *DIO3*-FA27 promoter

#### Primers’ creation and synthesis

**Fig. 1:**
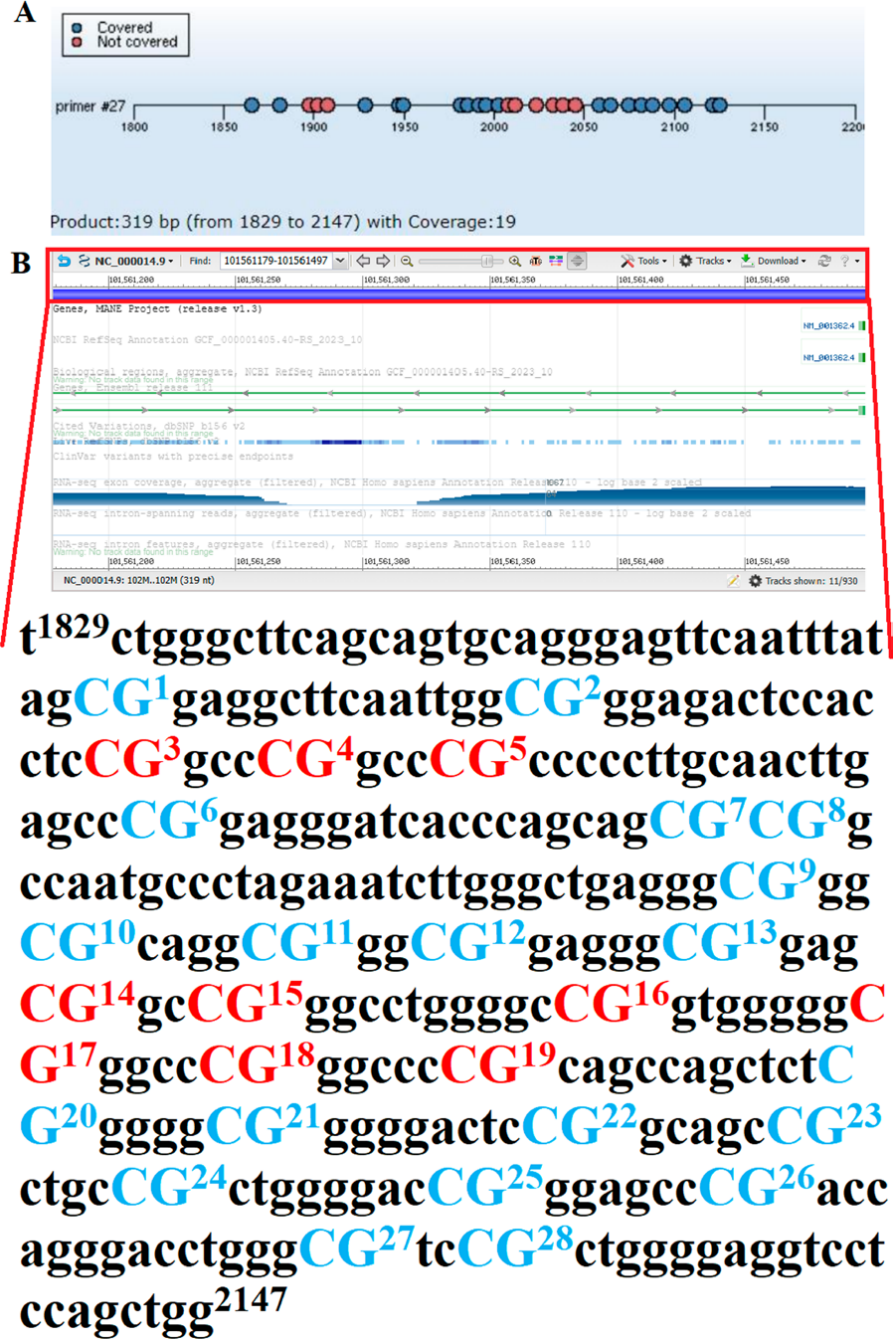
The information of CpG methylation sites in primer *DIO3*-FA27 and the target fragment sequence in the *DIO3* promoter region (**A** The dots represent the CpG sites to be tested in the target fragment; **B** The position of the amplified segment of interest on the chromosome: chr14: 101561179–101561497 and the *DIO3*-FA27 target fragment contained 28 CpGs: the blue bases represent CpG sites to be tested; the red base represents the CpG site that was not detected)

The sequence of the promoter region of the RefSeq NM_001362 was obtained on the NCBI website (https://www.ncbi.nlm.nih.gov/gene/). Beijing Boao Jingdian Biotechnology Co., Ltd (Beijing, China) synthesized primers spanning CpGs for quantitative methylation analysis, which were built using Agena’s software (http://www.epidesigner.com/index. html). The sequences of the forward and reverse primers were as follows, respectively, (Fig. 1A): Fw: TCTGGGCTTCAGCAGTGCAGGGAGTTC and Rv: TCCGCTGGGGAGGTCCTCCAGCTGG. This primer is intended to amplify the 1829 bp-2147 bp region of the promoter region, which is 319 bp length and contains 28 CpG sites. (Fig. 1B). These sites can only be examined as a whole because DNaseA cleaves the U3′ end only in the rare cases where the sequence between two or more CpG sites does not contain U (corresponding to T of the template DNA). The methylation level is an average of these sites.

#### MALDI-TOF MS-based quantitative methylation

DNA from HF and control peripheral blood samples was extracted using the QIAamp DNA Mini Kit (QIAGEN). 100 ngDNA was extracted and detected by 0.8% agarose gel electrophoresis, the genomic DNA electrophoresis band was usually not less than 20 kb, the main band of electrophoresis was obviously not degraded, A260/A280 = 1.7–2.1, and the total amount should not be less than 2 μg. Adjust the concentration to 75 ng/μl for qualified DNA. The genomic DNA was treated with bisulfite using EZ-96 DNA methylation kit (ZYMO Research, Irvine, CA) according to the manufacturer’s instructions and amplified by methylation-specific polymerase chain reaction (MSP).

To measure the *DIO3* CpG methylation, the Agena MassARRAY platform (CapitalBio Corporation, Beijing, China) was utilized. It includes an RNA base-specific cleavage (Mass CLEAVE) and a matrix-assisted laser desorption/ionization time-of-flight (MALDI-TOF) mass spectrometer. Using a Mass ARRAY Compact System and a Spectro CHIP (Agena Bioscience, California, USA), quantitative methylation data were collected. The data were analyzed and shown using the EpiTYPER software version 1.0 (Agena Bioscience, California, USA).

### Statistical analysis

The presentation of quantitative data is given as mean ± standard deviation (SD). The Kolmogorov–Smirnov method was used to test all measurement data for normality or lognormality. Students’ t test results between HF and controls were compared whether the data fit a normal or lognormal distribution. In the absence of that, a Mann–Whitney test was selected. Using the lowest group as a reference, the methylation level quartile of the control group was used to split the study subjects into four groups (Q1, Q2, Q3, and Q4). If the data passed tests for equal variance and normalcy (lognormality), then a one-way ANOVA with the LSD multiple comparison tests was performed to evaluate the differences from more than two samples. A Kruskal–Wallis test was used in its place. The Chi-square test was used to compare the count data, which are expressed as a rate and composition ratio (%).The restricted cubic spline (RCS) was used to analyze the correlation of clinical trial markers of CpG methylation level of *DIO3*-FA27 in patients with HF. The statistical software SPSS 26.0 (SPSS Inc., Chicago, IL, USA) was used for all analyses. The receiver operator characteristic (ROC) curve was plotted, and the area under curve (AUC), Youden index, and cutoff values of single and combined indicators were calculated, and *P* < 0.05 was a statistically significant difference.

## Results

### Population characterization

The general characteristics were compared, and it revealed that gender, age, region, daily temperature, breathing, pulse, systolic blood pressure, and diastolic blood pressure exhibited no statistically significant difference between the HF group and control group. (*P* > 0.05, Table 1).

**Table 1:** Baseline characteristics of the study population

Variables	HF (*n* = 20)	Control (*n* = 20)	*P-*value
*Sex*			
Male (%)	10.00 (25.00)	13.00 (32.50)	0.337
Female (%)	10.00 (25.00)	7.00 (17.50)	
*Region*			
Urban (%)	15.00 (37.50)	13.00 (32.50)	0.490
Rural (%)	5.00 (12.50)	7.00 (17.50)	
Age (years)	65.5 (48.0, 70.8)	69.00 (59.80, 75.80)	0.291
Temperature (℃)	36.40 (36.3, 36.5)	36.50 (36.40, 36.60)	0.386
Breathing (/min)	20.00 (20.0, 20.0)	20.00 (19.30, 20.00)	0.548
Pulse (/min)	71.00 (66.0, 90.3)	81.50 (73.80, 85.50)	0.101
SBP (mmHg)	117.40 ± 15.54	120.30 ± 12.43	0.291
DBP (mmHg)	78.21 ± 10.33	72.05 ± 16.04	0.165

*SBP* systolic blood pressure, *DBP* diastolic blood pressure

The Chi-square test was utilized for sex and region. Nonparametric tests were conducted for non-normal data such as age, temperature, and breathing pulse. Two independent sample *t* tests were employed for normal data such as SBP and DBP. Sex and Region expressed as number (*n*); Age, Temperature, and Breathing pulse expressed as M (P25, P75); SBP and DBP expressed as $\overline{x }$±SD

### CpG methylation of *DIO3*-FA27 of HF

**Fig. 2:**
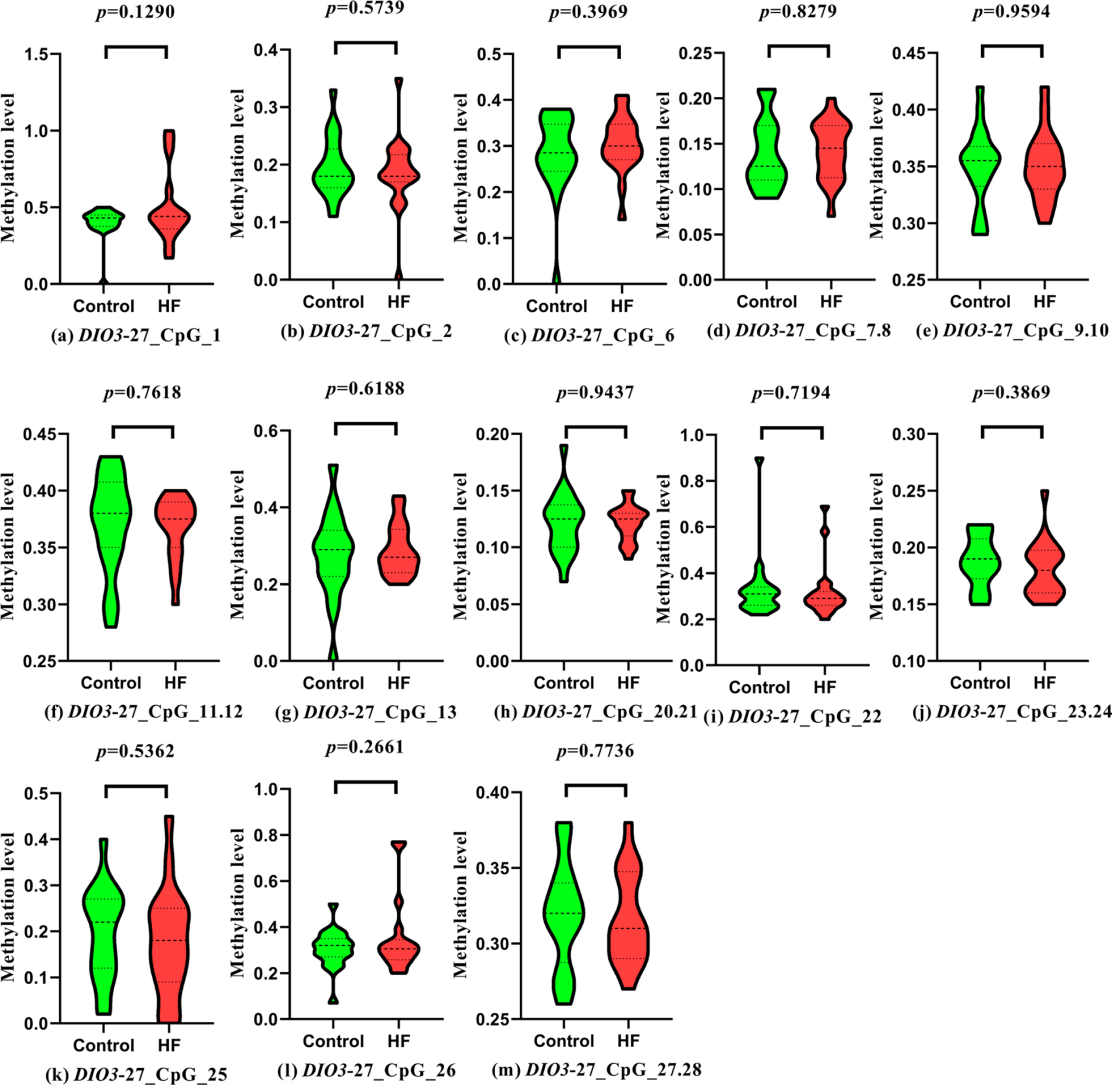
Comparison of methylation levels in the *DIO3*-FA27 promoter region between patients with HF and healthy controls

Using prism8.0 software, a violin plot comparing methylation levels of the promoter region of selenoprotein gene *DIO3*-FA27 in patients with HF and healthy control groups was fitted, indicating that a nonsignificant difference was observed in methylation fragments in the promoter region of *DIO3*-FA27 between patients with HF and healthy controls (*P* > 0.05, Fig. 2).

### Various degrees of CpG methylation of *DIO3*-FA27 in HF patients

**Fig. 3:**
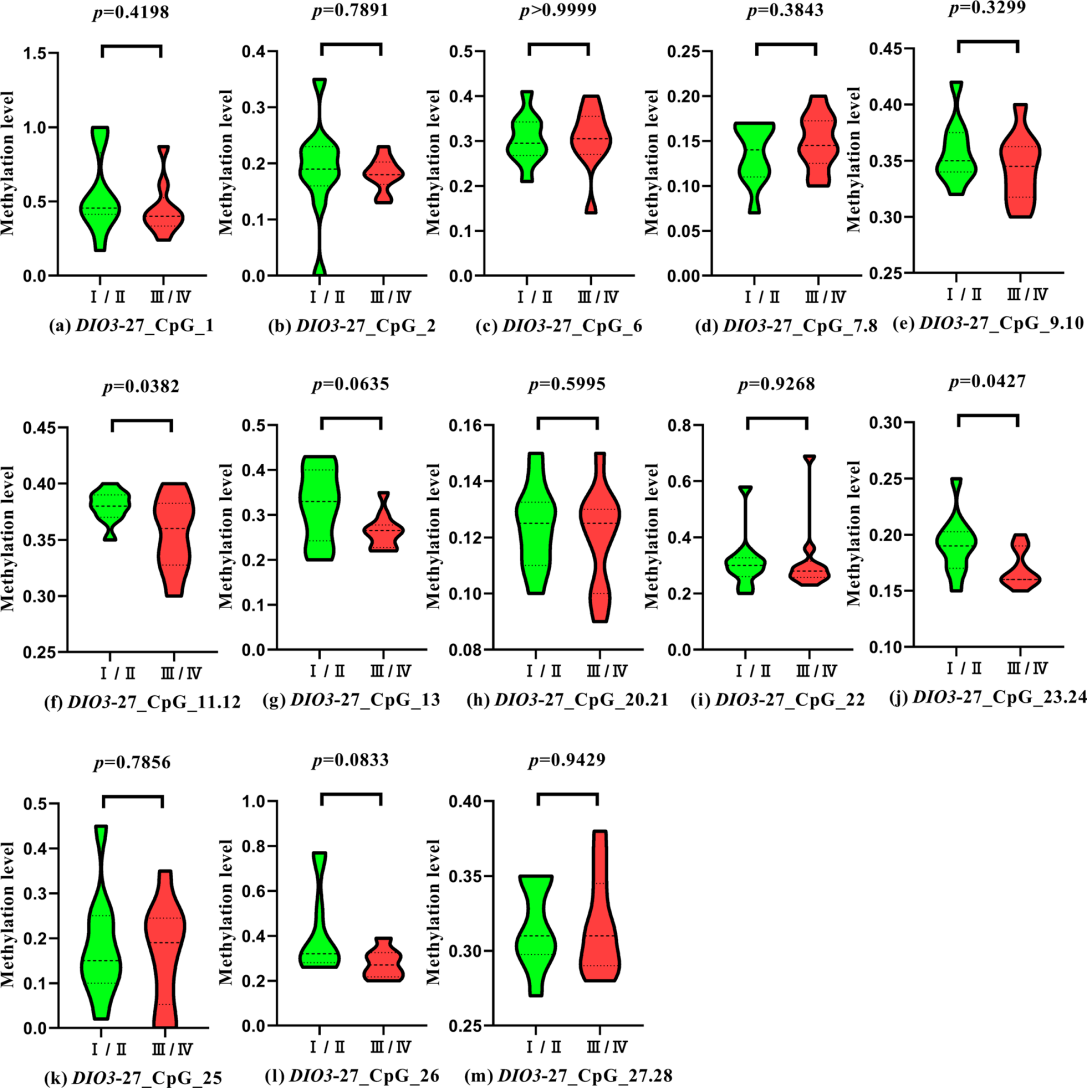
Comparison of methylation levels in the promoter region of *DIO3*-FA27 in patients with heart failure with different cardiac functions

The violin diagram results comparing methylation levels of *DIO3*-FA27 promoter region in patients with different HF degrees illustrated that in the heart function of patients with HF, the methylation fragments of the promoter region of the *DIO3*-FA27 promoter, *DIO3*-FA27_CpG_11.12 (*P* = 0.0382), and *DIO3*-FA27_CpG_23.24 (*P* = 0.0427) were statistically significant (*P* < 0.05, Fig. 3f–j).

### Analysis of influencing factors of clinical biochemical indices, CpG methylation, and HF

**Fig. 4:**
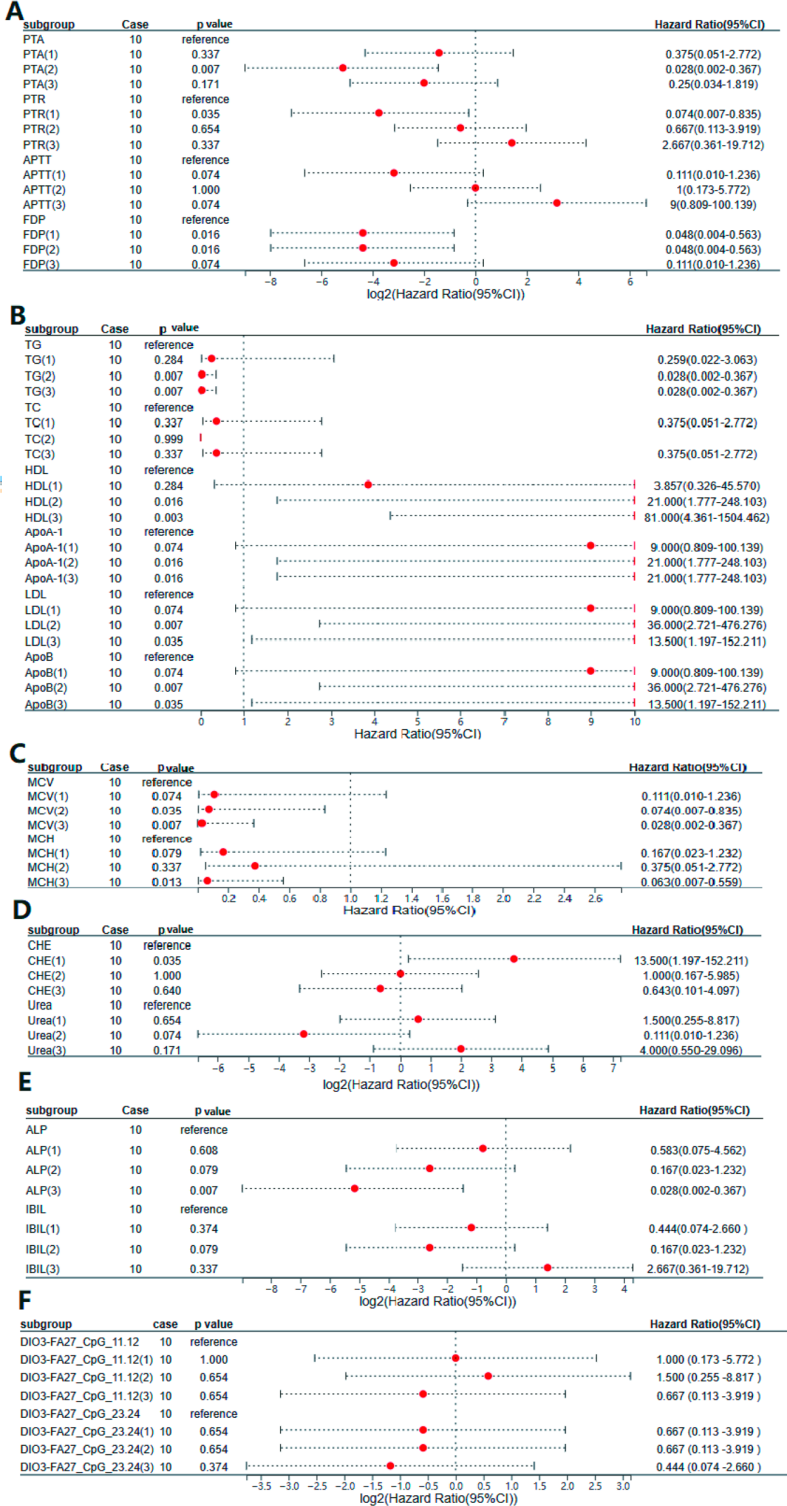
Univariate Logistic regression for HF. (**A** Coagulation index and HF. **B** Blood lipid index and HF. **C** Blood routine indices and HF. **D** Renal function indices and HF. **E** Liver function indices and HF. **F** *DIO3*-FA27 promoter region methylation tablets and HF)

Logistic regression was deployed to analyze the effects of various clinical biochemical indices on patients with HF, and the results were as follows:

Univariate logistic regression results manifested that triglyceride (TG), serum total cholesterol (TC), high-density lipoprotein (HDL), human Apolipoprotein A-I (ApoA-1), low-density lipoprotein (LDL), and ApoB were probably related to patients with HF. Meanwhile, prothrombin time activity (PTA), prothrombin time ratio (PTR), APTT, and fibrin degradation products (FDP) in coagulation indicators were probably correlated with HF. Among the renal function indices, cholinesterase (CHE) and urea were probably associated with patients with HF. Moreover, alkaline phosphatase (ALP) and indirect bilirubin (IBIL) in liver function indicators were probably related to patients with HF. Furthermore, mean corpuscular volume (MCV) and mean corpuscular hemoglobin concentration (MCH) were connected to patients with HF (*P* < 0.05). The effect of the methylation fragment of the promoter region of *DIO3*-FA27 on patients with HF represented a nonsignificant difference (*P* > 0.05, Fig. 4).

Therefore, the five items of blood lipids, coagulation, liver function, renal function, and blood routine in clinical biochemical indices, *DIO3*-FA27_CpG_11.12, and *DIO3*-FA27_CpG_23.24 may be related to HF.

In the analysis of differences between different cardiac function grades and biochemical indices, we can find that there are statistically significant differences in PTR and ApoA-1 between cardiac function I/II and III/IV. (*P* < 0.05, Table 2).

**Table 2:** Comparison of biochemical indices with different cardiac functions $({\bar{x}\pm{\text{SD}}})$

Biochemical Indices	I/II (*n* = 10)	III/IV (*n* = 10)	*P-*value
PTA (sec.)	2.60 ± 1.35	1.70 ± 0.95	0.10
PTR (sec.)	2.10 ± 1.29	3.40 ± 0.97	0.02
APTT (sec.)	2.90 ± 1.20	2.90 ± 1.37	1.00
FDP (mg/L)	1.80 ± 1.14	2.60 ± 1.35	0.17
TG (mmol/L)	1.80 ± 1.03	1.90 ± 0.99	0.83
TC(mmol/L)	2.40 ± 1.43	2.0 ± 1.15	0.50
HDL (mmol/L)	3.50 ± 0.53	2.90 ± 1.10	0.14
ApoA-1 (g/L)	3.60 ± 0.51	2.40 ± 0.84	0.01
LDL (mmol/L)	3.30 ± 0.67	2.60 ± 0.96	0.08
ApoB (g/L)	3.30 ± 0.67	2.60 ± 0.97	0.08
MCV (fL)	2.40 ± 0.97	1.50 ± 0.97	0.05
MCH (pg)	2.50 ± 1.08	1.70 ± 0.95	0.09
CHE (μ/L)	2.50 ± 0.97	2.10 ± 0.99	0.38
Urea (mmol/L)	2.10 ± 1.20	3.10 ± 1.20	0.08
ALP (μ/L)	1.70 ± 0.67	2.10 ± 1.10	0.34
IBIL (μmol/L)	2.90 ± 1.1	2.3 ± 1.50	0.32

*PTA* prothrombin time activity, *PTR* prothrombin time ratio, *APTT* activated partial thromboplastin time, *FDP* fibrin degradation products, *TG* triglyceride; *TC* serum total cholesterol; *HDL* high-density lipoprotein; *ApoA-1* apolipoprotein A-I, *LDL* high-density lipoprotein, *ApoB* apolipoprotein B; *MCV* mean volume of red blood cells, *MCH* mean corpuscular hemoglobin, *CHE* cholinesterase; *ALP* alkaline phosphatase, *IBIL* indirect bilirubin

The multivariate logistic regression results (Table 3) elucidated that APTT levels had significant differences (*P* < 0.05) in the Q3 (OR = 27.00, 95% CI 1.08–674.12) and Q4 groups (OR = 264.04, 95% CI 3.76–18548.42), which indicated that APTT was positively linked to patients with HF after the APTT level increased to Q3 group. Meanwhile, FDP levels were significantly different (*P* < 0.05) in the Q2 (OR = 0.00, 95% CI 0.00–0.09), Q3 (OR = 0.02, 95% CI 0.0–0.57), and Q4 groups (OR = 0.03, 95% CI 0.00–0.90), revealing that FDP level has a negative connection to patients with HF. A positive correlation existed between urea and patients with HF in the Q3 group (OR = 0.11, 95% CI 0.01–1.24). In the blood routine index, MCV was positively associated with patients with HF at the levels of Q3 and Q4 groups (OR = 0.07, 95% CI 0.00–0.84; OR = 0.03, 95% CI 0.00–0.37); HDL was positively linked to patients with HF in Q3 and Q4 groups (OR = 21.00, 95% CI 1.78–248.10; OR = 81.00, 95% CI 4.36–1504.46). In liver function indices, ALP was positively related to patients with HF at the Q4 group level (OR = 0.03, 95% CI 0.00–0.37).

**Table 3:** Multivariate logistic regression analysis of the influence of five biochemical markers on patients with HF

Variables	Case	Group	*β*-value	SE	*P-*value	OR	95% CI
*Coagulation indicators*							
APTT	10	Q1	Reference	–	–	–	–
	10	Q2	− 1.17	1.66	0.48	0.31	0.01–8.05
	10	Q3	3.30	1.64	0.04	27.00	1.08–674.12
	10	Q4	5.58	2.17	0.01	264.04	3.76–18,548.4
FDP	10	Q1	Reference	–	–	–	–
	10	Q2	− 6.65	2.15	0.00	0.00	0.00–0.09
	10	Q3	− 3.95	1.72	0.02	0.02	0.00–0.57
	10	Q4	− 3.49	1.72	0.04	0.03	0.00–0.90
*Renal indicators*							
Urea	10	Q1	Reference	–	–	–	–
	10	Q2	0.41	0.90	0.65	1.50	0.26–8.82
	10	Q3	− 2.20	1.23	0.07	0.11	0.01–1.24
	10	Q4	1.39	1.01	0.17	4.00	0.55–29.10
*Blood routine indicators*							
MCV	10	Q1	Reference	–	–	–	–
	10	Q2	− 2.20	1.23	0.07	0.11	0.01–1.24
	10	Q3	− 2.60	1.24	0.04	0.07	0.00–0.84
	10	Q4	− 3.58	1.32	0.00	0.03	0.00–0.37
*Lipid indicators*							
HDL	10	Q1	Reference	–	–	–	–
	10	Q2	1.35	1.26	0.28	3.86	0.33–45.57
	10	Q3	3.05	1.26	0.02	21.00	1.78–248.10
	10	Q4	4.40	1.49	0.00	81.00	4.36–1504.46
*Liver indicators*							
ALP	10	Q1	Reference	–	–	–	–
	10	Q2	− 0.54	1.05	0.61	0.58	0.08–4.56
	10	Q3	− 1.80	1.02	0.08	0.17	0.02–1.23
	10	Q4	− 3.39	1.32	0.08	0.03	0.00–0.37

### Dose–response relationship between HF differential CpG site methylation level and various clinical biochemical indices

Taking the 25th, 50th, and 75th percentiles of methylation levels at HF differential CpG sites as three nodes, a restrictive cubic spline model was established for eight clinical biochemical indices: blood pressure, blood lipid, coagulation, liver function, renal function, protein, electrolytes, and blood routine. However, it was found that the methylation levels of HF differential CpG sites *DIO3*-FA 27_CpG_11.12 and *DIO3*-FA 27_CpG_23.24 were only associated with coagulation, liver function, and kidney function. There exists a correlation and dose–response relationship between the four indices of blood routine.

#### HF differential CpG site methylation level in relation to dose–response of coagulation indices

**Fig. 5:**
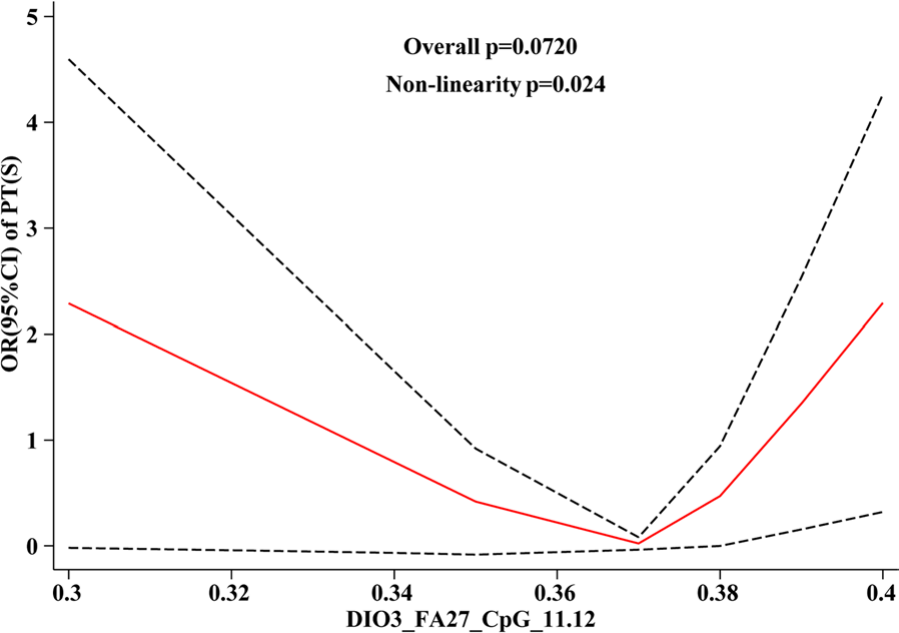
Relationship between HF differential CpG site methylation level and coagulation index

Using restricted cubic spline regression, the dose–response curves of methylation levels at *DIO3*-FA27_CpG_11.12 and *DIO3*-FA 27_CpG_23.24 sites on coagulation indices were further fitted. The solid line in Fig. 5 represents the OR of the coagulation index. The results (Fig. 5) illustrate that a correlation exists between plasma PT, PTA, PTR, partially activated APTT, fibrinogen degradation product FDP, and *DIO3*-FA27_CpG_11.12 site methylation level where PT, PTA, PTR, and FDP have all “U-shaped” nonlinear correlations with *DIO3*-FA27_CpG_11.12. However, no dose–response relationship exists (*P*_overall_ > 0.05, *P*_non-linnear_ < 0.05, Fig. 5A, B, C, and D).

The APTT and *DIO3*-FA27_CpG_11.12 had an “inverted U-shaped” nonlinear association, but no dose–response relationship was observed (*P*_overall_ > 0.05, *P*_non-linnear_ < 0.05, Fig. 5E).

#### HF differential CpG site methylation level in relation to dose response of liver function

**Fig. 6:**
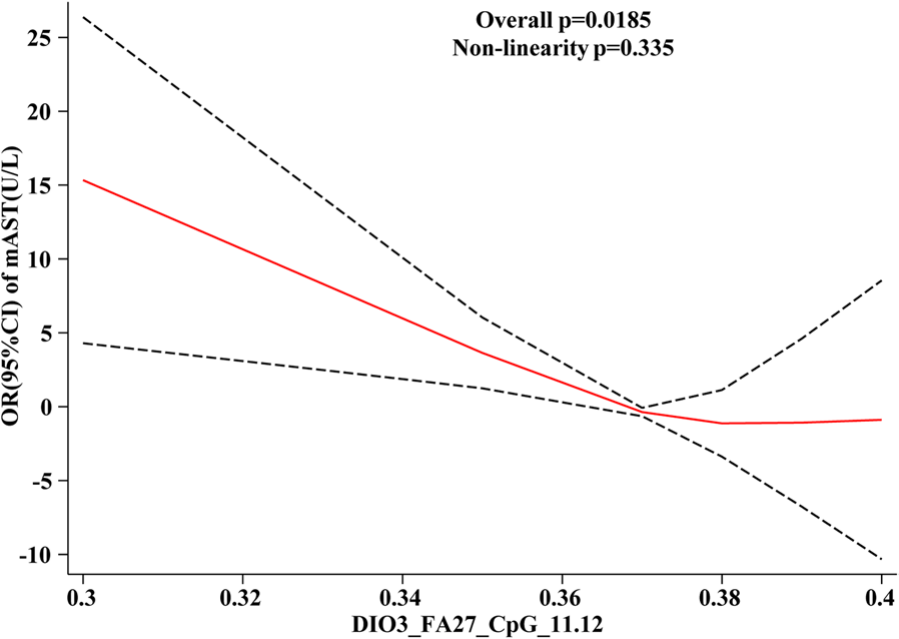
Dose–response relationship between HF differential CpG site methylation level and liver function indices

The dose–response relationship between the methylation levels of *DIO3*-FA27_CpG_11.12 and *DIO3*-FA27_CpG_23.24 sites on liver function is observed, and the solid lines in Fig. 6 represent the OR values of each liver function index. The analysis results (Fig. 6) revealed that mAST and TBA were associated with *DIO3*-FA27_CpG_11.12, both exhibiting a “U-shaped” linear dose–response relationship (*P*_overall_ < 0.05, *P*_non-linnear_ > 0.05), where mAST and *DIO3*-FA27_CpG_11.12 had a negative linear correlation. With the elevated methylation level at this site, the mAST OR value decreased (Fig. 6A).

The TBA was positively and linearly correlated with the methylation level of *DIO3*-FA27_CpG_11.12 site. With the escalated methylation level of this site, the OR of TBA also increased (Fig. 6B).

#### HF differential CpG site methylation level corresponding to dose response of renal function

**Fig. 7:**
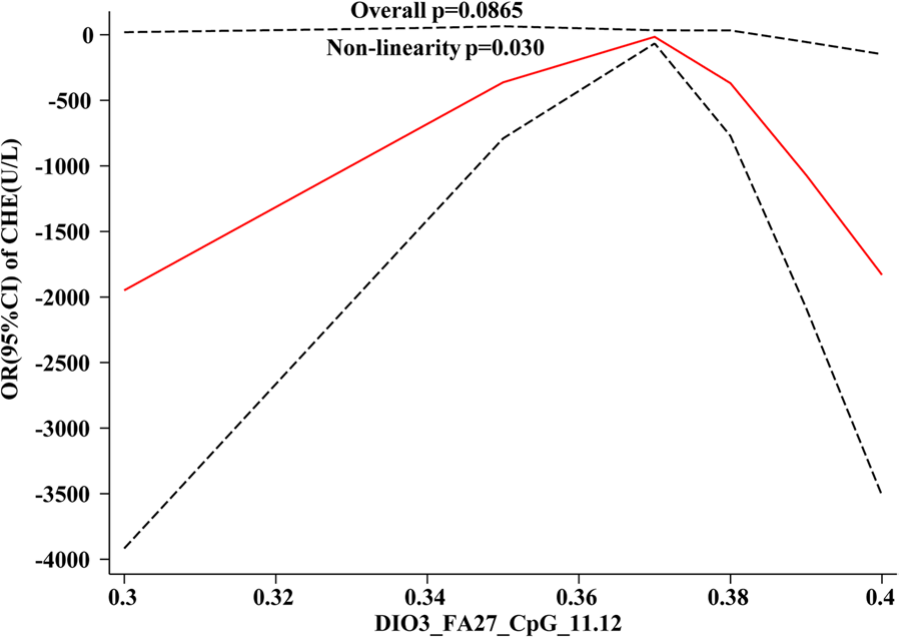
Dose–response relationship between HF differential CpG site methylation level and renal function indices

The dose–response relationship between the methylation levels of *DIO3*-FA27_CpG_11.12 and *DIO3*-FA27_CpG_23.24 sites on renal function (Fig. 7) indicated that CHE and CO_2_ were associated with *DIO3*-FA27_CpG_11.12 methylation levels, where CHE and the methylation level of this site demonstrated an “inverted U-shaped” nonlinear correlation, without dose–response relationship (*P*_overall_ > 0.05, *P*_non-linnear_ < 0.05). Meanwhile, CO_2_ revealed a “U-shaped” nonlinear association with methylation levels at this site but without a dose–response relationship (*P*_overall_ > 0.05, *P*_non-linnear_ < 0.05, Fig.7A, B).

#### HF differential CpG site methylation level corresponding to dose response of blood routine indices

**Fig. 8:**
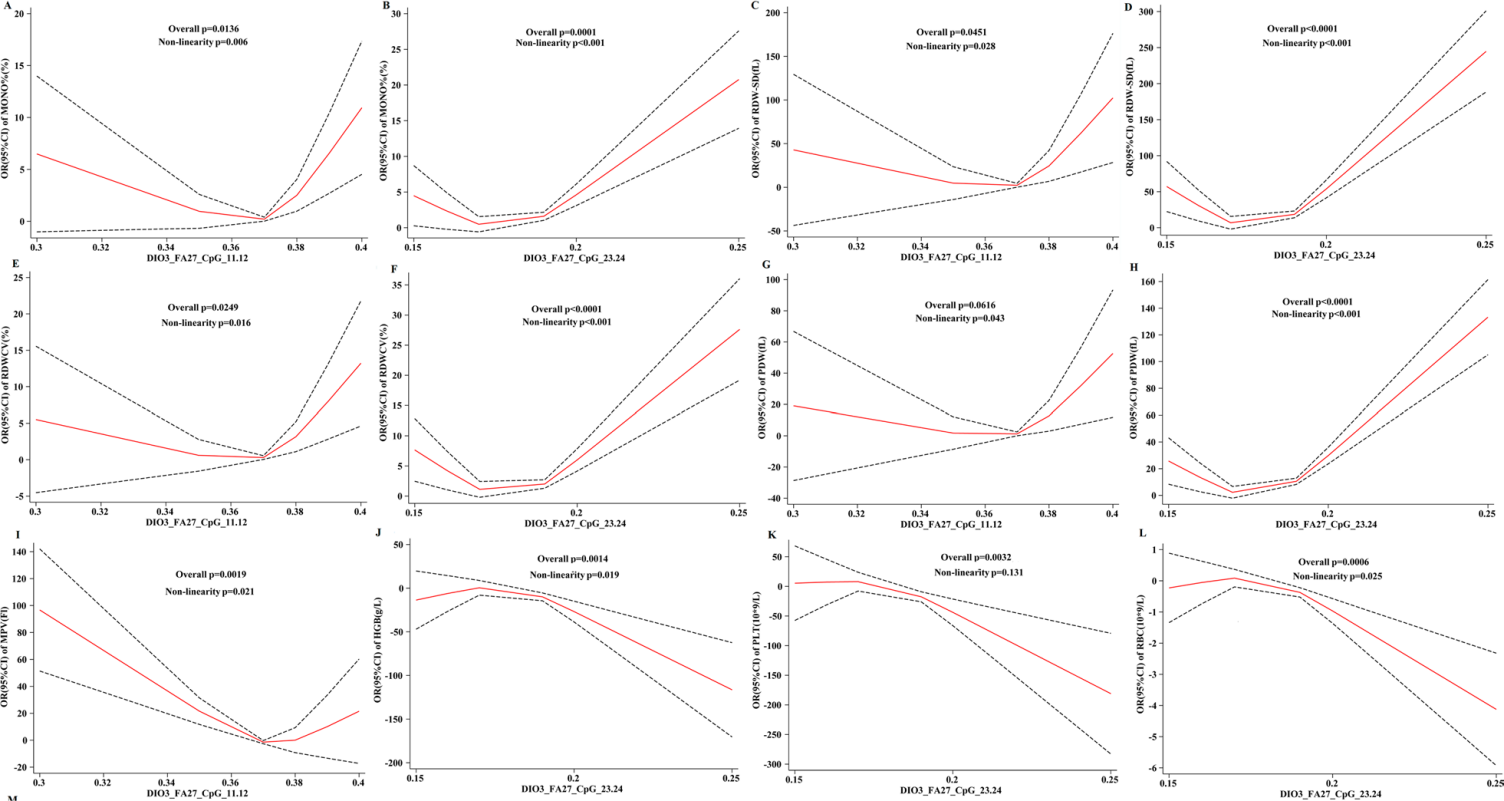
Relationship between HF differential CpG site methylation level and dose–response of blood routine 1 index

The dose–response relationship between methylation levels of *DIO3*-FA27_CpG_11.12 and *DIO3*-FA27_CpG_23.24 on routine 1 indices (Fig. 8) indicated that MONO%, RDW-SD, RDW-CV%, *DIO3*-FA27_CpG_11.12, and *DIO3*-FA27_CpG_23.24 exhibited “U-shaped” nonlinear associations with dose–response relationship (*P*_overall_ < 0.05, *P*_non-linnear_ < 0.05). Therefore, with the heightened *DIO3*-FA27_CpG_11.12 and *DIO3*-27_CpG_23.24 methylation levels, the OR of MONO%, RDW-SD, and RDW-CV% gradually decreased and then gradually increased, (Fig.  8A–F).

PDW was nonlinearly associated with the methylation level of *DIO3*-FA27_CpG_11.12 sites but without a dose–response relationship (*P*_overall_ > 0.05, *P*_non-linnear_ < 0.05). Furthermore, PDW exhibited a “U-shaped” nonlinear association with the *DIO3*-FA27_CpG_23.24 methylation level, with a dose–response relationship (*P*_overall_ < 0.05, *P*_non-linnear_ < 0.05). As the methylation level of this site elevates, the OR of PDW first gradually decreases and then gradually increases (Fig. 8G, H).

The MPV was nonlinearly associated with *DIO3*-FA27_CpG_11.12 as a “U-shaped,” with a dose–response relationship (*P*_overall_ < 0.05, *P*_non-linnear_ < 0.05). With the elevated methylation level of this site, the OR of MPV first gradually decreases and then gradually increases (Fig.  8I).

**Fig. 9:**
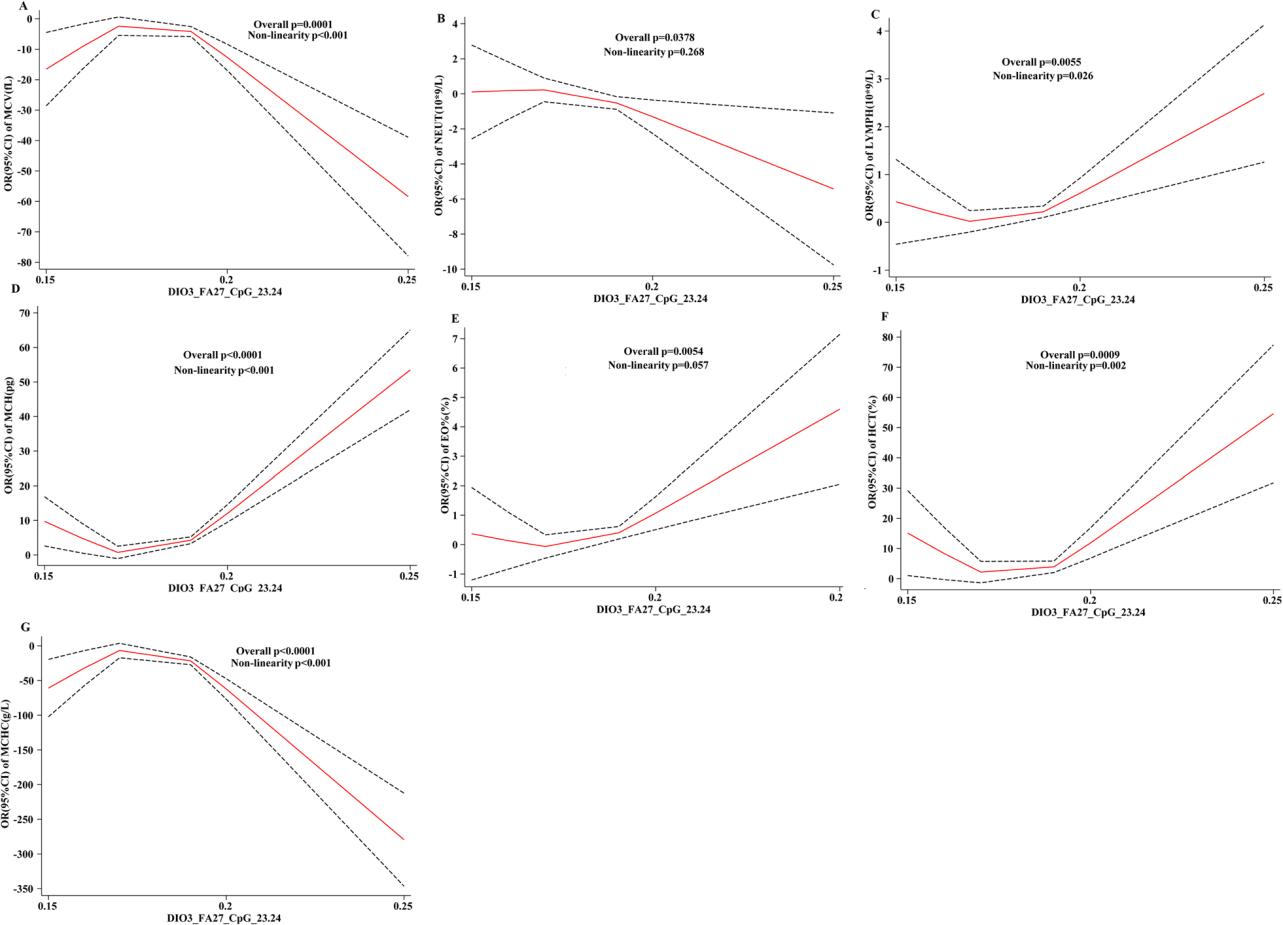
Relationship between HF differential CpG site methylation level and dose–response of blood routine 2 index

The dose–response relationship between HF differential CpG sites *DIO3*-27_CpG_11.12 and *DIO3*-FA27_CpG_23.24 in routine 2 revealed the following results: RBC, PLT, HGB, NEUT, HCT, MCHC, MCV, and *DIO3*-FA27_CpG_23.24 showed a nonlinear association of “inverted U-shaped” and a dose–response relationship (*P*_overall_ < 0.05, *P*_non-linnear_ < 0.05). Briefly, with the elevated methylation level of this site, the OR of RBC, PLT, HGB, NEUT, HCT, MCHC, and MCV gradually increased to a certain value and then gradually decreased (Fig. 8J-L, Fig. 9A, B, F, G).

Moreover, LYMPH, MCH, EO%, and *DIO3*-FA27_CpG_23.24 methylation levels all exhibited “U-shaped” nonlinear associations, with a dose–response relationship (*P*_overall_ < 0.05, *P*_non-linnear_ < 0.05). The results indicated that with the raised methylation levels at this site, the OR of LYMPH, MCH, and EO% gradually decreased and then increased (Fig. 9C–E).

According to the results of RCS, these four types of clinical biochemical indices: coagulation indices (PT, PTA, PTR, APTT, and FDP), liver function indicators (mAST and TBA), renal function indicators (CHE and CO_2_), blood routine indicators (MONO%, RDW-CV, MPV, HGB, PLT, RBC, PDW, RDW-SD, NEUT, EO%, MCV, MCH, MCHC, HCT, and LYMPH), and HF have a dose–response relationship. Results of RCS model with *P*＞0.05 for other clinical biochemical indices with methylation levels of the key CpGs are attached to Additional file 1.

### ROC analysis for coagulation, liver function, renal function, and blood routine diagnosis of HF

**Fig. 10:**
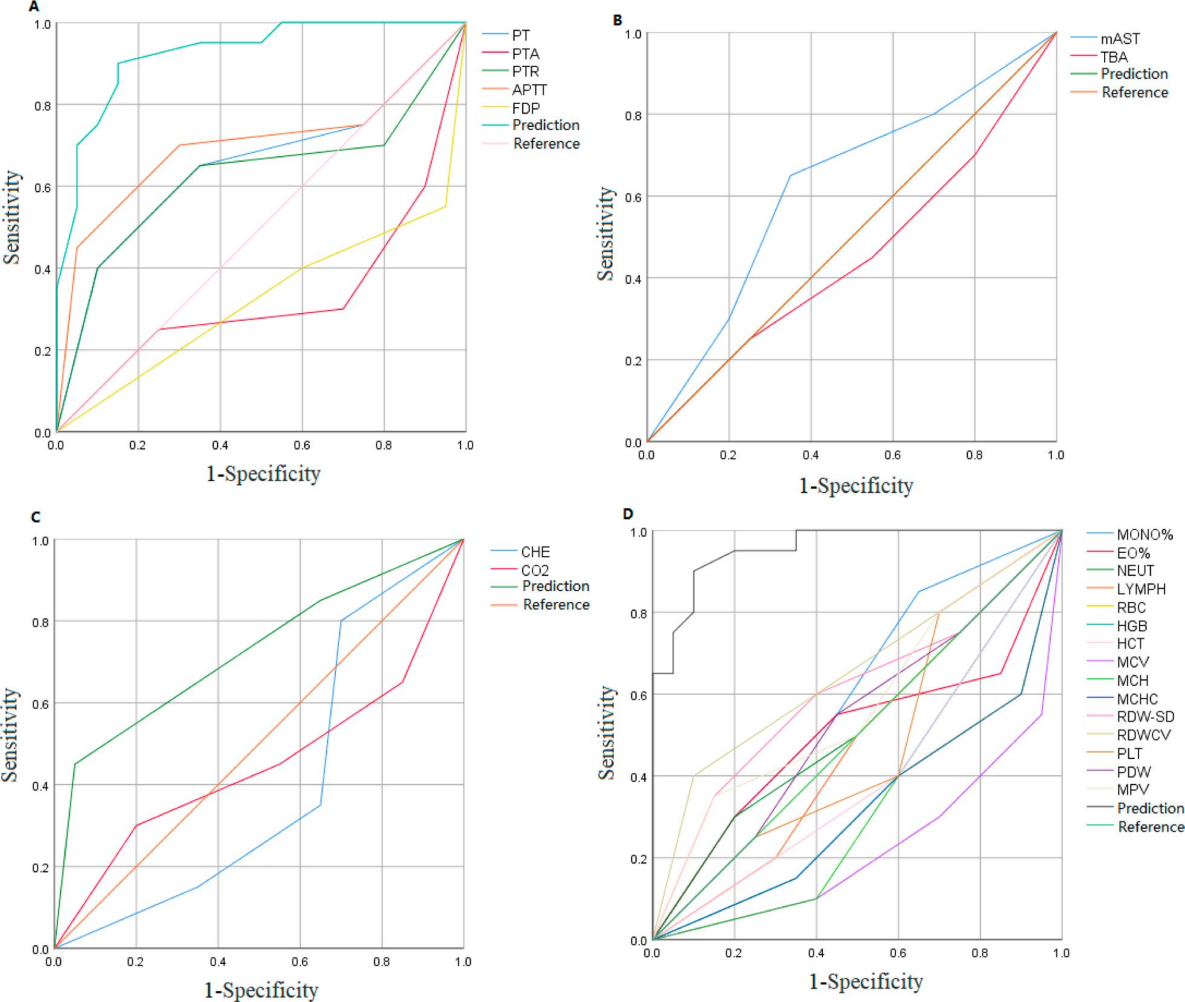
ROC curves of four types of clinical biochemical indices for HF diagnosis (**A** ROC curve of coagulation index combined diagnosis of HF. B ROC curve of liver function indices combined to diagnose HF. **C** ROC curve of renal function indices combined for the diagnosis of HF. **D** ROC curve for combined diagnosis of HF by blood routine indices)

According to the RCS results, the ROC curves of coagulation, liver function, kidney function, and blood routine indices were established, and the results showed that the AUC (95% *CI*) of the four biochemical index models for diagnosing HF was 0.93 (0.84–1.00), 0.50 (0.32–0.68), 0.73 (0.57–0.88), and 0.96 (0.90–1.00). The blood routine index combined with HF was the best among the four indicators (Table 4 and Fig. 10).

**Table 4:** ROC curve parameters of four biochemical markers

Variables	AUC	95% CI	Sensitivity	Specificity	Youden index	*P*-value
Coagulation model	0.93	0.84–1.00	0.90	0.85	0.75	< 0.01
Liver model	0.50	0.32–0.68	0.65	0.65	0.30	1.00
Renal model	0.73	0.57–0.88	0.45	1.00	0.40	0.02
Blood routine model	0.96	0.90–1.00	0.90	0.90	0.80	< 0.01

## Discussion

There is evidence linking a number of selenoproteins to cardiovascular disease. The DIO’s main task is control thyroid hormone levels, which reduce the remodeling of the heart following myocardial infarction [15]. HF is linked to lower thyroid hormone plasma levels and higher *DIO3* expression. The results demonstrated that *DIO3* can prevent congenital cardiac defects in a mouse model of comprehensive developmental thyrotoxicosis related to a lack of *DIO3*, highlighting the protective effect of *DIO3* during development [16, 17]. Consequently, this study analyzed the differences between *DIO3*-FA27 methylation fragments and biochemical indices in patients with HF to discover their role in the condition and provide a theoretical basis for future HF diagnosis and treatment.

Recent research suggests that by altering the epigenome, micronutrients like selenium may modify the risk of disease and have an impact on health outcomes [18]. The underlying mechanisms are believed to result from selenium’s influence on the three unique and intimately related systems that control gene expression throughout the life course: histone modification, DNA methylation, and non-coding microRNAs [19]. DNA methyltransferases (DNMTs) are a class of biological enzymes whose activity directly affects the methylation status of DNA [20, 21]. It has been found that after knocking out the methyltransferase DNMT3b in the heart of adult mice, it was found that the myocardial contractility of mice was weakened, the myocardium became thinner, and the myocardial fibers and muscle nodules were disordered, which could lead to a decline in cardiac function and eventually heart failure, and the methylation levels of DNMT3b promoter and CpG island in myocardial tissue were significantly reduced compared with the normal control group [22]. In an in vivo and in vitro study to evaluate the effects of selenium supplementation on induced cardiac dysfunction, selenium supplementation was found to significantly improve induced cardiac systolic and diastolic dysfunction by inhibiting oxidative stress-mediated cardiomyocyte apoptosis. Selenium supplementation restores antioxidant balance, thereby inhibiting cardiac oxidative stress and promoting DNA methylation in the promoter region of the selenium gene by reducing DNMT2 [23].

This study found nonsignificant fragments in the difference analysis of methylation fragments in the *DIO3*-FA27 promoter region between the healthy and heart failure groups. However, in the heart failure group, the difference analysis between I/II and III/IV revealed that *DIO3*-FA27_CpG_11.12 and *DIO3*-FA27_CpG_23.24 had statistical significance. There is strong evidence that atherosclerotic lesions in cardiovascular disease have altered globally in terms of genomic methylation [24]. One such instance is the unexpected activation of *DIO3* in the heart during ventricular remodeling and HF, which has been shown in multiple animal models [25].

The methylation levels of *DIO3*-FA27 CpG were different between the HF group and the control group, and also differed in the cardiac function I/II and III/IV groups in this study. The RCS analysis demonstrated that a dose–response relationship existed between PT, PTA, MONO%, MPV, HGB, PLT, RBC, PDW, HCT, and LYMPH, among others, and methylation sites. When these biochemical indices escalated to a certain extent, it elevated heart failure risk. The comprehensive results indicated that combining biochemical indices and DNA methylation of the *DIO3*-FA27 promoter may increase the risk of grade in HF.

It was discovered that a number of laboratory indices, including as SCR, BUN, glucose, and APTT, were useful indicators of heart failure [26]. Herein, we discussed whether biochemical indices had an impact on HF. Our results revealed that coagulation index APTT, renal function index urea, blood lipid index HDL, and liver function index ALP were positively related to HF when elevated to a certain level, while FDP levels had a negative connection. Furthermore, the effectiveness of the common coagulation routes as well as the “intrinsic” (now known as the contact activation pathway) is measured by PTT or APTT, a performance indicator. In contrast, prolonged APTT indicates Von Willebrand disease or Hemophilia. Conversely, It has been established that the severity of coronary heart disease in relation to HF may be significantly predicted by the plasma levels of Von Willebrand disease [27]. Accordingly, it has been established that PTT and APTT can predict HF.

Numerous in vivo instances have demonstrated that biochemical indices could alter global DNA methylation [28]. Higher plasma Hcy levels, for instance, have been linked to increased AdoHcy concentrations and lymphocyte DNA hypomethylation in healthy individuals [29]. Other findings have corroborated the inverse relationship between Hcy plasma concentrations and DNA methylation patterns, which has been expanded to include multiple animal models [30].

The measure of platelet size and activity, or MPV, has been linked favorably to an increased risk of cardiovascular disease and is thought to be an inflammatory biomarker in the cardiovascular system [31, 32]. According to published studies, there is a substantial correlation between raised MPV and the higher prevalence and severity of atherosclerotic cardiovascular disease [33]. This study found a dose–response relationship between biochemical indicators such as MPV and before heart failure, consistent with previous results.

This study performed ROC analysis on biochemical indices and demonstrated that blood routine has certain predictive power for heart failure. A small number of common test findings can distinguish between HF predictions that are similar to those of more complex and costly biomarkers including PTX3, brain natriuretic peptide (BNP), NT-proBNP, H-FABP, and mid-regional pro-adrenomedullin (MR-proADM)ADDIN. Furthermore, there are shortcomings in blood routine indicator detection, such as the absence of detection for B-type cerebral urine natriuretic peptide indicators. Biochemical indicators of heart failure have proven efficient in diagnosing heart failure, and natriuretic peptides, particularly in establishing HF diagnosis. It has been determined that left ventricular systolic dysfunction (LVSD) is associated with elevated levels of BNP and its N-terminal precursor, N-terminal proBNP (N-BNP), which has high negative predictive values for LVSD diagnosis [34].

We were able to analyze 5′methylcytosine at the genomic level, which allowed us to discover the relationship between methylation levels, biochemical indices, and HF patients, something that traditional genomics studies could not find. However, our study also has some limitations. The first limitation is that the sample size is relatively small and selection bias cannot be completely ruled out. Second, we did not measure the levels of *DIO3* protein in this study, which requires further research. More studies are needed in the future to confirm the correlation between *DIO3*-FA27 promoter methylation, biochemical indices, and HF progression. And the intervention in biochemical indices level is expected to improve the long-term prognosis of patients with HF.

Finally, we examined the biochemical indices and CpG methylation profiles in the *DIO3* promoter area in blood samples from HF patients and healthy controls. The *DIO3*-FA27 promoter DNA methylation showed hypomethylation in HF patients with different grades, and its biochemical indices combined with certain levels of *DIO3*-FA27 promoter DNA methylation could heighten the risk of grade in patients with HF. Meanwhile, the combination of blood routine indices was found to be the most effective in diagnosing patients with HF. This study provides a new perspective on HF pathogenesis.

## Conclusions

The *DIO3*-FA27 promoter DNA methylation showed hypomethylation and biochemical indicators in HF patients with different levels. Higher APTT, HDL, and MPV levels, among others, combined with certain *DIO3*-FA27 promoter DNA methylation levels, can increase the risk of worsening severity classification in patients with HF. Moreover, the combination of blood routine indicators has a superior ability to diagnose HF.

### Supplementary Information

**Additional file 1: Fig. S1.** Dose-response relationship between HF related differential methylated CpGs and biochemical indicators.

## Data Availability

Data will be available upon request from the corresponding author.

## Abbreviations

HF: Heart failure
PT: Prothrombin time
PTA: Prothrombin time activity
PTR: Prothrombin time ratio
APTT: Activated partial thromboplastin time
FDP: Fibrin degradation products
mAST: Mitochondrial aspartate aminotransferase
TBA: Total bile acids
CHE: Cholinesterase
CO_2_: Carbon dioxide
MONO%: Monocytes ratio
RDW-CV: Red blood cell distribution width-coefficient of variation
MPV: Mean platelet volume
HGB: Hemoglobin
PLT: Platelet
RBC: Red blood cell count
PDW: Platelet distribution width
RDW-SD: Red blood cell distribution width-standard deviation
NEUT: Neutrophil
EO%: Eosinophil ratio
MCV: Mean volume of red blood cells
MCH: Mean corpuscular hemoglobin
MCHC: Mean corpuscular hemoglobin concentration
HCT: Hematocrit
LYMPH: Lymphocyte
DIO: Deiodinase
MALDI-TOF-MS: Matrix-assisted laser desorption/ionization time-of-flight mass spectrometry
TBIL: Total bilirubin
PT: Prothrombin time
APTT: Activated partial thromboplastin time
BUN: Blood urea nitrogen
SCR: Serum creatinine
TG: Triglyceride
TC: Serum total cholesterol
HDL: High-density lipoprotein
ApoA-1: Apolipoprotein A-I
LDL: Low-density lipoprotein
ALP: Alkaline phosphatase
IBIL: Indirect bilirubin
