# Supplementary material for: Evaluating the link between DIO3-FA27 promoter methylation, biochemical indices, and heart failure progression
Source: Clin Epigenetics. 2024 Apr 24;16:57. doi: 10.1186/s13148-024-01668-0 (PMC11040988; doi:10.1186/s13148-024-01668-0)

Additional file:

Fig. S1. Relationship between HF differential CpG site methylation level and dose-response of biochemical indices.


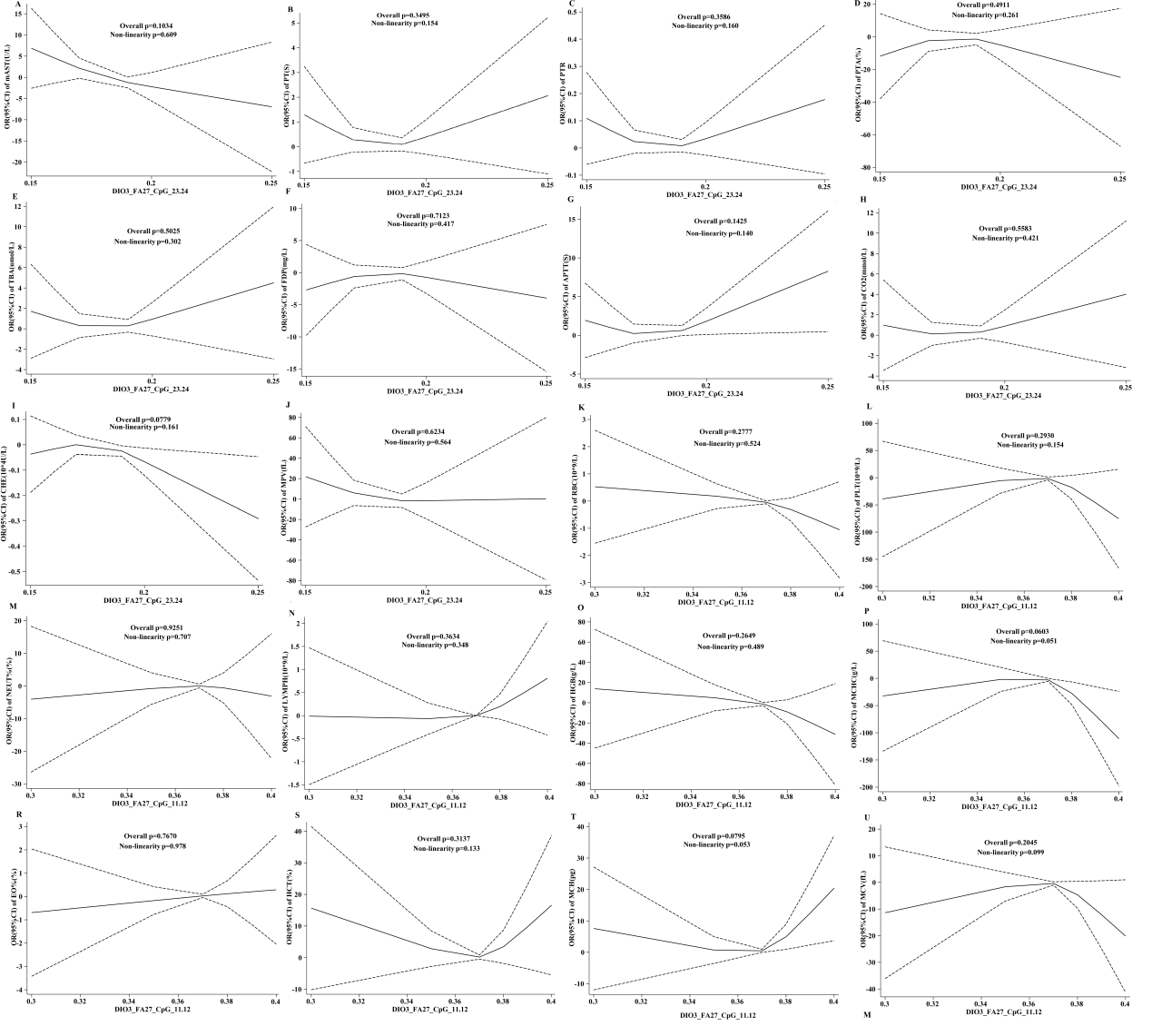

Supplement: Supplementary file 1 — Additional file 1: Fig. S1. Dose-response relationship between HF related differential methylated CpGs and biochemical indicators. [file 13148_2024_1668_MOESM1_ESM.docx]
